# La proteína de unión a los ácidos grasos cardíaca (HFABP) está relacionada con la gravedad de la insuficiencia cardíaca y sus biomarcadores cardíacos conocidos

**DOI:** 10.1515/almed-2021-0071

**Published:** 2021-11-10

**Authors:** Damien Gruson, Christina Adamantidou, Sylvie A. Ahn, Michel F. Rousseau

**Affiliations:** Centro de investigación en Endocrinología, Diabetes y Nutrición, Instituto de Investigación Experimental y Clínica, Cínicas universitarias St-Luc y Universidad Católica de Louvain, Bruselas, Belgica; Departamento de Bioquímica Clínica, Cínicas universitarias St-Luc y Universidad Católica de Louvain, Bruselas, Belgica; División de Cardiología, Cínicas universitarias St-Luc y Centro de Investigación Cardiovascular, Instituto de Investigación Experimental y Clínica, Cínicas universitarias St-Luc y Universidad Católica de Louvain, Bruselas, Belgica

**Keywords:** biomarcador, insuficiencia cardíaca, NT-proBNP, proteína de unión a los ácidos grasos cardíaca (HFABP), resultado, riesgo

## Abstract

**Objetivos:**

Determinar las concentraciones de proteína de unión a los ácidos grasos cardíaca (HFABP) en pacientes con insuficiencia cardíaca con fracción de eyección reducida (ICFEr) y su potencial valor pronóstico.

**Métodos:**

Se determinaron las concentraciones circulantes de HFABP mediante un inmunoensayo quimioluminiscente automático en 25 voluntarios sanos y 60 pacientes con ICFEr.

**Resultados:**

Los pacientes con insuficiencia cardíaca (IC) presentaron concentraciones de HFABP significativamente mayores que los voluntarios sanos. Se observó una correlación significativa entre los niveles de HFABP, la clasificación de la New York Heart Association (NYHA), y las concentraciones de los biomarcadores de disfunción y remodelado cardíaco (NT-proBNP, FGF-23 y galectina-3). Las concentraciones de HFABP también mostraron valor predictivo de muerte cardiovascular, y su combinación con NT-proBNP podría ser sinérgica a la hora de evaluar el riesgo.

**Conclusiónes:**

Las concentraciones de HFABP están aumentadas en los pacientes con ICFEr, se relacionan con el riesgo cardiovascular y podrían ayudar a los especialistas en el manejo de los pacientes.

## Introducción

La insuficiencia cardíaca (IC) afecta a millones de personas en todo el mundo y está asociada a un mal pronóstico [1, 2]. Los péptidos natriuréticos, el péptido natriurético tipo B (BNP) y el pro-BNP amino-terminal (NT-proBNP), son biomarcadores habitualmente empleados en el diagnóstico y manejo de pacientes con IC [3, 4].

El hallazgo de nuevos biomarcadores procedentes de otras vías fisiopatológicas podría mejorar la estratificación del riesgo, el manejo de resultados y la selección de tratamiento para los pacientes con IC [1, 5, 6]. Por esta razón, se ha analizado el valor añadido de los biomarcadores relacionados con el remodelado y la fibrosis cardíaca, como el ST2 soluble, la galectina-3 o el factor de crecimiento de fibroblastos 23 (FGF-23), así como de los biomarcadores de necrosis miocárdica como la troponina [7, 8].

Se ha estudiado la relación de la proteína de unión a los ácidos grasos cardíaca (HFABP) en multitud de patologías, y esta se ha relacionado con la detección temprana de la isquemia, útil para el diagnóstico temprano del infarto de miocardio [9, 10]. El aumento de HFABP también se ha asociado con la IC, y su determinación podría aportar información suplementaria en la estratificación del riesgo de los pacientes con IC [9, 10].

El objetivo de este estudio es determinar las concentraciones de HFABP en pacientes con IC con fracción de eyección reducida (ICFEr) y evaluar su valor pronóstico.

## Materiales y métodos

En nuestro estudio se incluyeron 25 voluntarios sanos sin tratamiento médico y sin antecedentes de hipertensión, diabetes, enfermedad renal crónica o enfermedades cardiovasculares, y 60 pacientes con IC con fracción de eyección ventricular izquierda reducida (ICFEr, fracción de eyección (FE) inferior al 35%). El estado funcional de los pacientes con IC se determinó según los criterios de la New York Heart Association (NYHA), identificando a 24 pacientes con ICC moderada (NYHA II) y a 36 con ICC grave (NYHA III-IV). En 47 pacientes, la IC fue debida a una miocardiopatía isquémica, presentando el resto miocardiopatía dilatada. Como criterio principal de valoración, se estableció la muerte cardiovascular en un periodo de 3,8 años. Todos los pacientes firmaron el consentimiento informado. El estudio fue aprobado por el comité ético correspondiente.

La HFABP y la troponina fueron determinadas en el analizador Maglumi^®^ 800 (Snibe diagnostics, Shenzhen, China) mediante inmunoensayo de quimioluminiscencia basado en el marcador amino-butil-etil-isoluminol (ABEI). ABEI es una pequeña molécula no enzimática con una fórmula molecular especial, que mejora la estabilidad de las soluciones ácidas y alcalinas.

El proceso de reacción química de ABEI, en el que se emplea hidróxido de sodio (NaOH) y peróxido de hidrógeno (H_2_O_2_), finaliza en tres segundos. Se midieron las concentraciones de NT-proBNP en muestras séricas mediante inmunoensayo de quimioluminiscencia en la plataforma Cobas^®^ 8000 (Roche Diagnostics, Mannheim, Germany). El coeficiente de variación interensayo de la troponina I observado en nuestro laboratorio fue del 5,8% para una concentración de 5,3 ng/L. Se validó localmente una concentración de 10 ng/L en el percentil 90 para los sujetos sanos.

Para determinar las concentraciones de galectina-3 (BG Medicine, Waltham, MA, USA) y los fragmentos C-terminales de FGF-23 (Immutopics, San Clemente, CA, USA) se utilizó el ensayo por inmunoadsorción ligado a enzimas (ELISA).

### Análisis estadístico

La normalidad en la distribución de las variables se evaluó mediante la prueba de Shapiro-Wilks. Cuando fue necesario, se realizó la transformación logarítmica de los datos, previamente al análisis estadístico. Las diferencias entre los voluntarios sanos y los pacientes con IC se evaluaron mediante análisis de varianza de una vía con la prueba de Student-Newman-Keuls para su comparación por pares. Se calcularon los coeficientes de correlación de Spearman para analizar las relaciones entre los biomarcadores. La influencia de la edad, la FE y los biomarcadores en la supervivencia se evaluó mediante el análisis univariante de riesgo proporcional de COX. Se estimó la curva de supervivencia de los pacientes con respecto a la mediana de HFABP y se realizó la comparación mediante la prueba de Mantel-Cox. El poder de discriminación entre biomarcadores se calculó mediante el análisis de la curva ROC, con el criterio definido de muerte cardiovascular al final del periodo de seguimiento. Los valores de p<0,05 se consideraron estadísticamente significativos. El análisis estadístico se realizó con el programa informático Medcalc.

## Resultados

**Figura 1:**
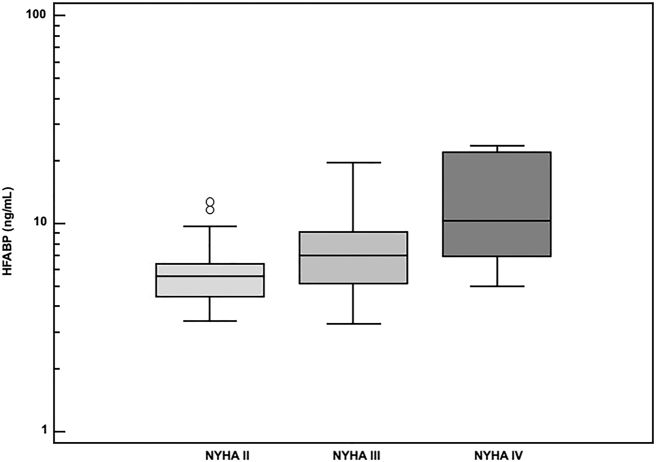
Niveles circulantes de HFABP en relación con las clases de la New York Heart Association (NYHA). HFABP, proteína de unión a los ácidos grasos cardíaca.

El grupo de pacientes con IC estaba compuesto por 15 mujeres y 45 hombres. La media de edad fue de 69,5 años, mientras que la FE media fue de 22,3%. Las concentraciones de HFABP fueron significativamente mayores en los pacientes con IC (mediana: 6,3 ng/mL; rango: 3,3–23,6 ng/mL) frente a los voluntarios sanos (2,2 ng/mL; 0,3–4,5). Se observó una relación significativa entre los niveles de HFABP y las clases funcionales de la NYHA (p<0,001), siendo las medias geométricas 5,6 ng/mL en NYHA II, 7,1 ng/mL en NYHA III y 11,1 ng/mL en NYHA IV (Figura 1). Las concentraciones medias de NT-proBNP, troponina I, galectina-3 y FGF-23 fueron de 4517 ng/L, 29,8 ng/L, 18,5 ng/mL y 346 RU/mL, respectivamente. Se observaron correlaciones significativas entre las concentraciones de HFABP, NT-proBNP, la troponina I, la galectina-3 y el FGF-23 (Tabla 1).

**Tabla 1:** Matríz de correlación entre HFABP y marcadores cardíacos en pacientes con IC grave.

	HFABP, pg/mL	NT-proBNP, pg/mL	Troponina I, ng/mL	Galectina-3, pg/mL	FGF-23, ng/L	
HFABP, pg/mL	–	0,56	0,25	0,68	0,48	r
		<0,001	0,05	0,41	<0,001	valor p
NT-proBNP, pg/mL	0,56	–	0,43	0,50	0,67	r
	<0,001		<0,001	0,04	<0,001	valor p
Troponina I, ng/mL	0,25	0,43	–	0,14	0,94	r
	0,05	<0,001		0,29	0,009	valor p
Galectina-3, pg/mL	0,68	0,50	0,14	–	0,56	r
	0,41	0,04	0,29		<0,001	valor p
FGF-23, ng/L	0,48	0,67	0,94	0,56	–	r
	<0,001	<0,001	0,009	<0,001		valor p

**Figura 2:**
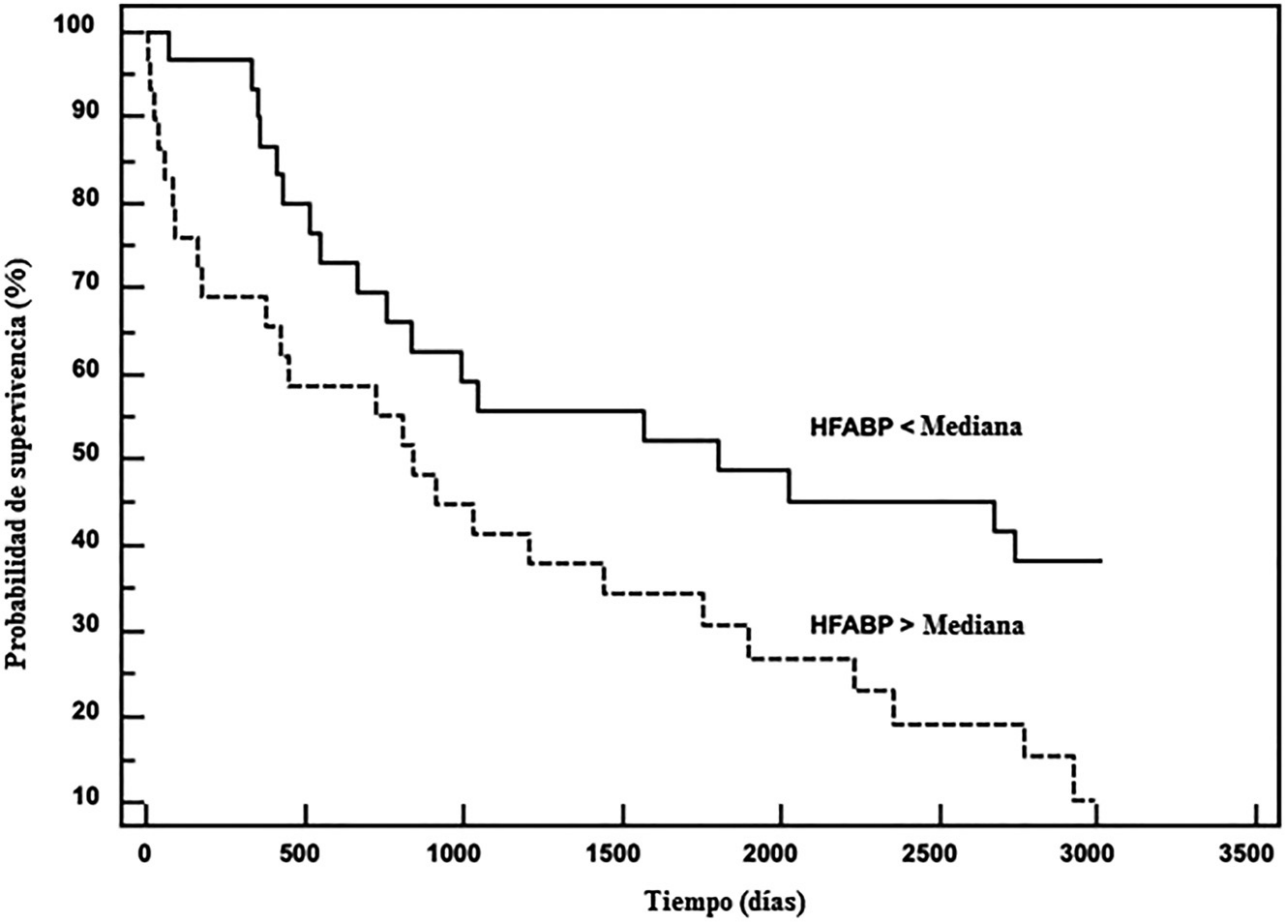
Curvas de supervivencia Kaplan-Meier en pacientes con IC grave, en relación con la mediana de HFABP. IC, insuficiencia cardiaca; HFABP, proteína de unión a los ácidos grasos cardíaca.

Con un seguimiento medio de 3,8 años, 43 pacientes con IC fallecieron (exacerbación de la IC, n=28; muerte súbita, n=10; otros tipos de muerte CV, n=5) y 4 pacientes se sometieron a un trasplante de corazón. La regresión de COX reveló una relación estadísticamente significativa entre los niveles de HFABP y la muerte cardiovascular (p=0,016), siendo significativamente divergentes las curvas Kaplan-Meier de los pacientes estratificados según la concentración media de HFABP (prueba de Mantel-Cox: p = 0,024, Figura 2). El área bajo la curva ROC fue inferior para HFABP, 0,63 (IC95%: 0,53–0,72), comparado con NT-proBNP, 0,74 (0,65–0,82) pero significativamente mayores que para la troponina I, 0,50 (0,37–0,63). Sin embargo, cuando se combinaron NT-proBNP y HFABP en una estrategia multimarcador, la tasa de muerte CV al final del seguimiento fue del 46% en los pacientes con IC y con dos biomarcadores por debajo de la mediana (n=18), 69% en los pacientes con IC y con uno de los biomarcadores superior a la mediana (n=18); y 88% en los pacientes con IC y con los dos biomarcadores superiores a la mediana (n=24). Así, postulamos que, en una estrategia multimarcador, la determinación de HFABP podría aportar un valor añadido de alrededor del 20% con respecto al análisis de NT-proBNP, a la hora de estimar el riesgo en pacientes con IC.

## Discusión

Identificar el subfenotipo de ICFEr es importante para desarrollar una estratificación del riesgo personalizada y mejorar el manejo de los pacientes con IC, a lo que puede contribuir el análisis de biomarcadores. Nuestros resultados muestran una correlación positiva entre las concentraciones de HFABP y la gravedad de la IC. La HFABP también está relacionada con biomarcadores conocidos de remodelado cardíaco y con muerte cardiovascular a largo plazo.

Implementar nuevos biomarcadores relacionados con la IC en la clínica sigue resultando complicado, y los posibles candidatos aún tienen que demostrar un buen rendimiento analítico, valor clínico añadido y buena relación coste-efectividad [1, 6]. Nuestros hallazgos demuestran que durante la evolución de la ICFEr se libera HFABP, ya que se observó un incremento de los niveles de HFABP según aumentaba la gravedad de la ICFEr. También hallamos una intensa correlación positiva de HFABP con la galectina-3 y el FGF-23, dos biomarcadores relacionados con el remodelado cardíaco y, más específicamente, con la inflamación cardiovascular, la fibrosis y la hipertrofia [6, 11]. Los resultados obtenidos concuerdan con estudios recientes, que muestran una correlación estadísticamente significativa entre las concentraciones de HFABP y los parámetros ecocardiográficos de remodelado ventricular izquierdo, y con un mal pronóstico en los pacientes con IC descompensada [12]. Los resultados también muestran que los niveles de HFABP están asociados a un mayor riesgo de muerte cardiovascular, ya que las curvas Kaplan-Meier identificaron un cambio en la supervivencia basado en la mediana de HFABP.

Nuestros resultados coinciden con la literatura actual. De hecho, en estudios previos ya se habían observado niveles elevados de HFABP en la insuficiencia cardíaca con fracción de eyección normal [13]. Existe evidencia de que los pacientes con disfunción ventricular izquierda asintomática muestran niveles de troponina T de alta sensibilidad y HFABP superiores a los voluntarios sanos. En otro estudio reciente, se observó que la medida adicional de HFABP mejoraba la especificidad diagnóstica y el valor predictivo positivo del análisis de NT-proBNP solo [14]. Las concentraciones altas de HFABP se asociaron a un mayor riesgo de muerte a los 5 años, lo cual coincide con nuestras observaciones.

Los datos muestran también que, en la estrategia de múltiples biomarcardores, el análisis de HFABP aportó un valor añadido a la prueba de NT-proBNP para la estratificación del riesgo en pacientes con IC. Estas estrategias que combinan varios biomarcadores son actualmente posibles, gracias a la automatización del HFABP y la determinación simultánea de NT-proBNP, pudiendo contribuir en la identificación de aquellos pacientes con ICFEr con mayor riesgo de muerte cardiovascular, lo que podría ayudar a personalizar el manejo de la enfermedad y la selección de tratamiento.

La integración de datos es también prometedora, gracias al uso de la inteligencia artificial y a la oportunidad que ello brinda de combinar los datos clínicos, los biomarcadores y los parámetros ecocardiográficos, lo que podría mejorar la precisión diagnóstica, la estratificación del riesgo y las decisiones clínicas [15].

Cabe señalar que nuestro estudio presenta algunas limitaciones, como el bajo número de pacientes, lo que impide realizar un análisis por rangos intercuartílicos o analizar la relación entre los biomarcadores y la disfunción sistólica y diastólica.

En conclusión, existe una correlación entre las concentraciones de HFABP y la gravedad de la ICFEr, habiéndose hallado también una asociación entre la HFABP y marcadores conocidos de la enfermedad, así como con un mayor riesgo cardiovascular. La determinación conjunta de HFABP y NT-proBNP podría ser sinérgica a la hora de identificar el subfenotipo de los pacientes con ICFEr, pudiendo contribuir a una atención más personalizada.

## References

[1] Seferovic PM, Ponikowski P, Anker SD, Bauersachs J, Chioncel O, Cleland JGF (2019). Clinical practice update on heart failure 2019: pharmacotherapy, procedures, devices and patient management. An expert consensus meeting report of the Heart Failure Association of the European Society of Cardiology. Eur J Heart Fail.

[2] Groenewegen A, Rutten FH, Mosterd A, Hoes AW (2020). Epidemiology of heart failure. Eur J Heart Fail.

[3] Mueller C, McDonald K, de Boer RA, Maisel A, Cleland JGF, Kozhuharov N (2019). Heart Failure Association of the European Society of Cardiology practical guidance on the use of natriuretic peptide concentrations. Eur J Heart Fail.

[4] Ponikowski P, Voors AA, Anker SD, Bueno H, Cleland JGF, Coats AJS (2016). 2016 ESC Guidelines for the diagnosis and treatment of acute and chronic heart failure. Eur Heart J.

[5] Burns DJP, Arora J, Okunade O, Beltrame JF, Bernardez-Pereira S, Crespo-Leiro MG (2020). International consortium for health outcomes measurement (ICHOM): standardized patient-centered outcomes measurement set for heart failure patients. JACC Heart Fail.

[6] Piek A, Du W, de Boer RA, Silljé HHW (2018). Novel heart failure biomarkers: why do we fail to exploit their potential?. Crit Rev Clin Lab Sci.

[7] Roy C, Lejeune S, Slimani A, de Meester C, Ahn As SA, Rousseau MF (2020). Fibroblast growth factor 23: a biomarker of fibrosis and prognosis in heart failure with preserved ejection fraction. ESC Heart Fail.

[8] Homsak E, Gruson D (2020). Soluble ST2: a complex and diverse role in several diseases. Clin Chim Acta.

[9] Rezar R, Jirak P, Gschwandtner M, Derler R, Felder TK, Haslinger M (2020). Heart-type fatty acid-binding protein (H-FABP) and its role as a biomarker in heart failure: what do we know so far?. J Clin Med.

[10] Otaki Y, Watanabe T, Kubota I (2017). Heart-type fatty acid-binding protein in cardiovascular disease: a systemic review. Clin Chim Acta.

[11] Fauconnier C, Roy T, Gillerot G, Roy C, Pouleur A-C, Gruson D (2019). FGF23: clinical usefulness and analytical evolution. Clin Biochem.

[12] Kazimierczyk E, Kazimierczyk R, Harasim-Symbor E, Kaminski K, Sobkowicz B, Chabowski A (2018). Persistently elevated plasma heart-type fatty acid binding protein concentration is related with poor outcome in acute decompensated heart failure patients. Clin Chim Acta.

[13] Dinh W, Nickl W, Füth R, Lankisch M, Hess G, Zdunek D (2011). High sensitive troponin T and heart fatty acid binding protein: novel biomarker in heart failure with normal ejection fraction? A cross-sectional study. BMC Cardiovasc Disord.

[14] Hoffmann U, Espeter F, Weiß C, Ahmad-Nejad P, Lang S, Brueckmann M (2015). Ischemic biomarker heart-type fatty acid binding protein (hFABP) in acute heart failure - diagnostic and prognostic insights compared to NT-proBNP and troponin I. BMC Cardiovasc Disord.

[15] Gruson D, Bernardini S, Dabla PK, Gouget B, Stankovic S (2020). Collaborative AI and Laboratory Medicine integration in precision cardiovascular medicine. Clin Chim Acta.

